# Fluoroscopy-Guided Removal of Tethered Surgical Drain via Guidewire Electrification: A Case Report

**DOI:** 10.7759/cureus.86923

**Published:** 2025-06-28

**Authors:** Austin Feng, Cordelia Orillac, Eytan Raz, Donato Pacione, Anthony Frempong-Boadu

**Affiliations:** 1 Neurosurgery, New York University (NYU) Langone Health, New York, USA; 2 Interventional Neuroradiology, New York University (NYU) Langone Health, New York, USA

**Keywords:** complications of spine surgery, guidewire electrification, spine surgery, surgical drains, tethered drain

## Abstract

Tethered postoperative drains are not uncommon complications that often require open removal in the operating room, which can increase risks of surgical site infection as well as length of hospitalization. We present a novel method of tethered drain removal through guidewire electrification. A retained deep drain following a posterior cervical laminectomy and fusion was identified after failed manual removal. Under fluoroscopic guidance, the retaining suture was indirectly identified through the obstruction of an inserted guidewire, through which monopolar cautery was applied, breaking the suture and allowing drain removal. The original incision did not need to be re-opened. While further investigation is necessary for validation, this technique shows great promise as an alternative to open removal.

## Introduction

Postoperative drains are important, especially in spine surgery, because they can prevent the development of hematoma, seroma, and infection and thereby help with wound healing [1-3]. While removal is usually uncomplicated, drains may be tethered in the wound from unintentionally being sutured in, and this issue is usually not able to be identified until the removal attempt. Tethered drain removal often requires return to the operating room (OR) for open removal, which causes increased perioperative pain for the patient, can increase risks of infection and wound healing issues, and can increase length of hospital stay [4,5]. As such, other avenues of retrieval are worthy of evaluation.

Attempts to force manual removal may dislodge the drain, but can lead to breakage and retention of a drain fragment, which would also require return to OR to remove the broken drain fragment [4,6]. Other methods have been described in case reports and series, such as percutaneous removal with fluoroscopic guidance, endoscopic release, as well as application of weighted traction to the drain [6-8]. In the present study, we present a proof of concept for the removal of tethered drain via cautery with a guidewire under fluoroscopic guidance.

## Case presentation

A 45-year-old female patient with prior surgical history of anterior cervical fusions presented for the removal of hardware and posterior cervical fusion, given persistent mechanical neck pain and hand numbness in the setting of pseudoarthrosis and failure of structured conservative therapy. She underwent a C4-C6 laminectomy with bilateral facetectomies/foraminotomies with C2-T2 posterior instrumented fusion. The wound was closed in layers, with 0-0 vicryl used for the muscle and fascia, 2-0 vicryl for the subcutaneous tissue, inverted 2-0 vicryl for the subdermal tissue, and running 4-0 monocryl for skin closure. Bilateral 15-French (Fr) Blake drains (right deep/left superficial) were also placed during closure. The patient's hospital course was uncomplicated, with drain outputs progressively decreasing daily. She was planned for removal of bilateral drains and discharge home on postoperative day 4. The superficial drain was removed without issue, but the deep drain could not be removed despite forceful pulling. The decision was made to bring the patient to a hybrid surgical suite to first attempt drain removal via guidewire cauterization.

**Figure 1:**
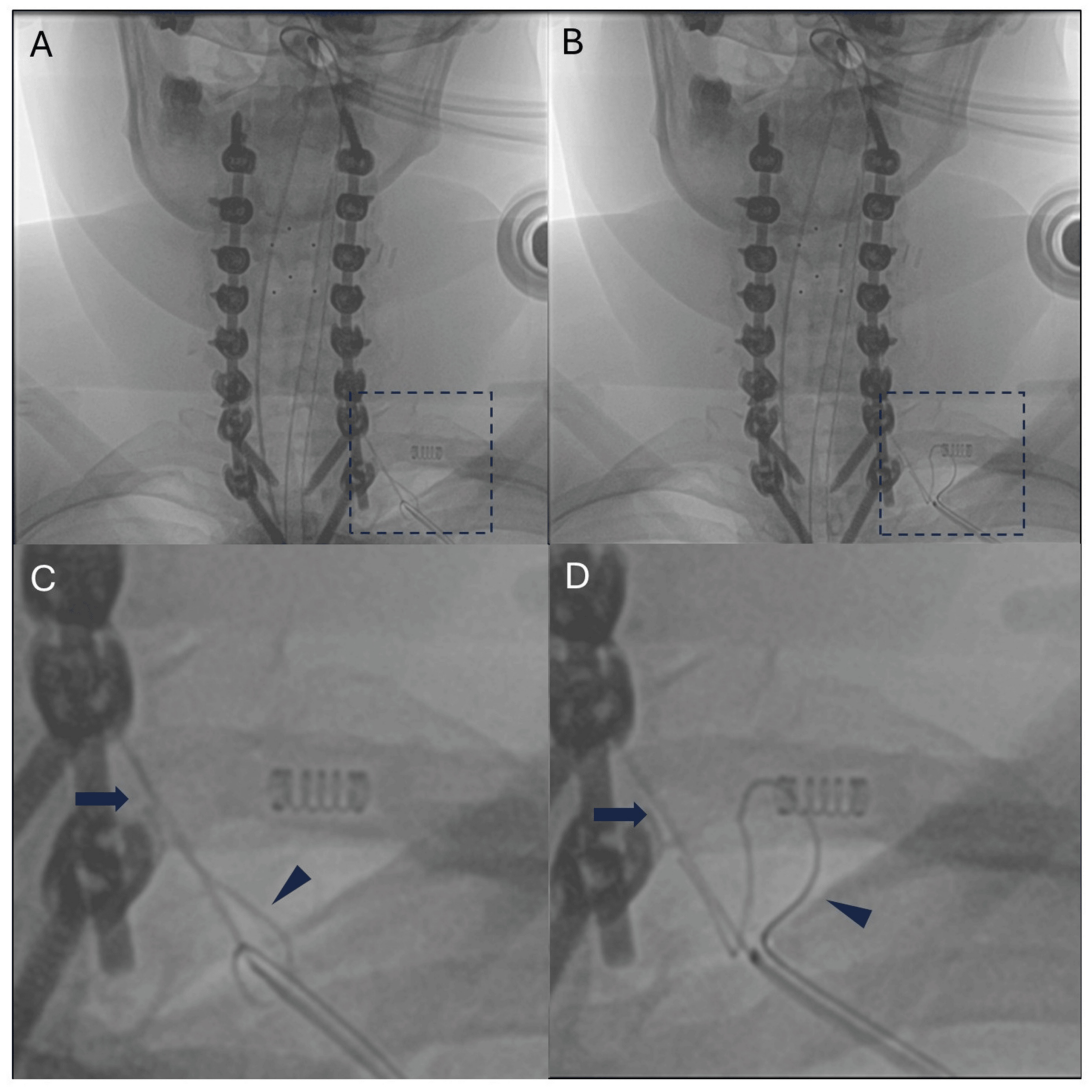
Fluoroscopic images of retained drain with inserted guidewires (A/B) Different attempts of passing a guidewire through the drain, with abnormal bending seen at the obstructed lumen while unhindered passage at an unobstructed lumen. Dotted boxes are enlarged to focus on the guidelines (C/D). The arrows point to the obstructed guidewire, bending due to the presumed tethering suture. In comparison, the arrowheads point to an unobstructed guidewire, which smoothly passed through the drain lumen. Of note, the springs seen in the middle of panels C and D are from clips on the patient and unrelated to the drain.

**Figure 2:**
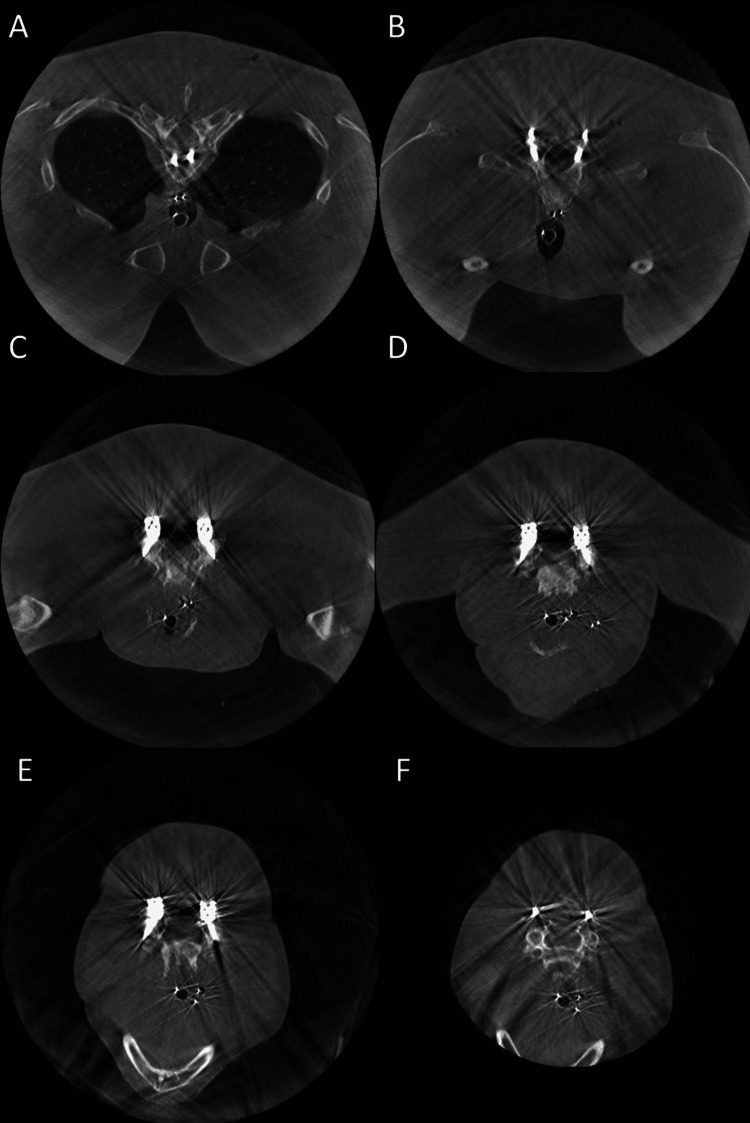
Dyna-CT demonstrating the removal of tethered drain without residual fragments

After intubation, the patient was positioned prone on the operating table. The external portion of the Blake drain was cut so that approximately an inch and a half of the drain was still visible above the skin. Of note, a Blake drain consists of four distinct lumens. The internal drain position was confirmed using biplanar intraoperative fluoroscopic imaging. Next, guidewires (Bentson straight fixed core (0.035-inch diameter) and spring wire with J-tip (0.021-inch diameter)) were advanced through the lumen of the drain until an obstruction was felt in one of the lumens. This was also confirmed using biplanar fluoroscopy (Figure 1). The obstructed guidewire was then electrified using monopolar cautery. After cauterization, the guidewire was advanced beyond the level of the obstruction easily, and the entire drain was then removed effortlessly. A Dyna-CT scan confirmed that there were no drain fragments inside the patient (Figure 2). The procedure was well tolerated by the patient. She was admitted to the recovery room in stable condition and discharged the following day.

## Discussion

Tethered drains can present a vexing challenge to surgeons. A return to the OR for removal not only exposes the patient to further surgical risks but also incurs increased healthcare costs. To the best of our knowledge and review of the literature, this is the first report of tethered drain removal via guidewire cauterization under fluoroscopic guidance.

**Figure 3:**
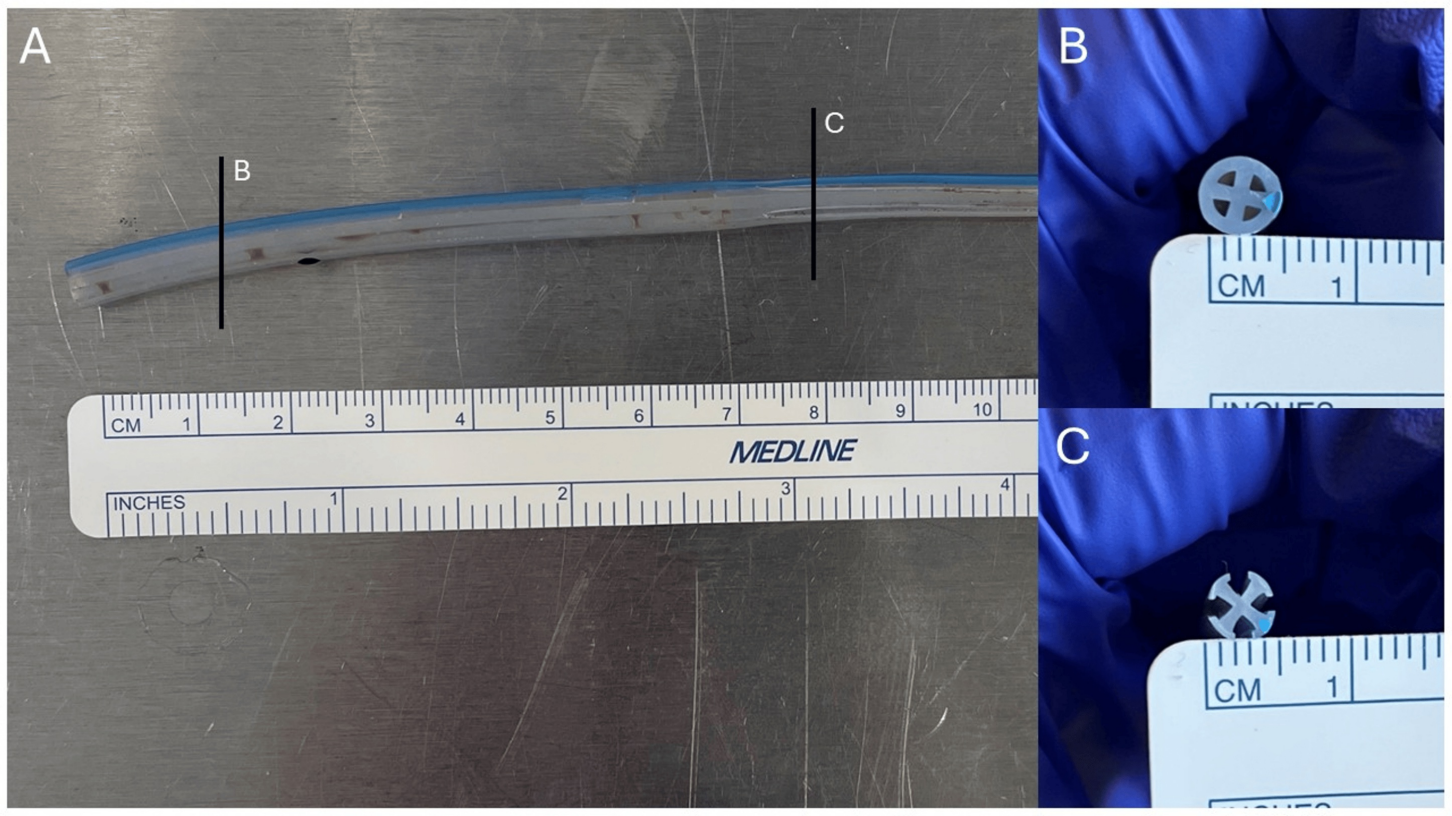
Removed tethered drain (A) Segment of the Blake drain. Cross-section of different portions displaced on side panels. (B) Distal portion of the Blake drain with four enclosed lumens. (C) Proximal portion of the Blake drain with four open lumens.

**Figure 4:**
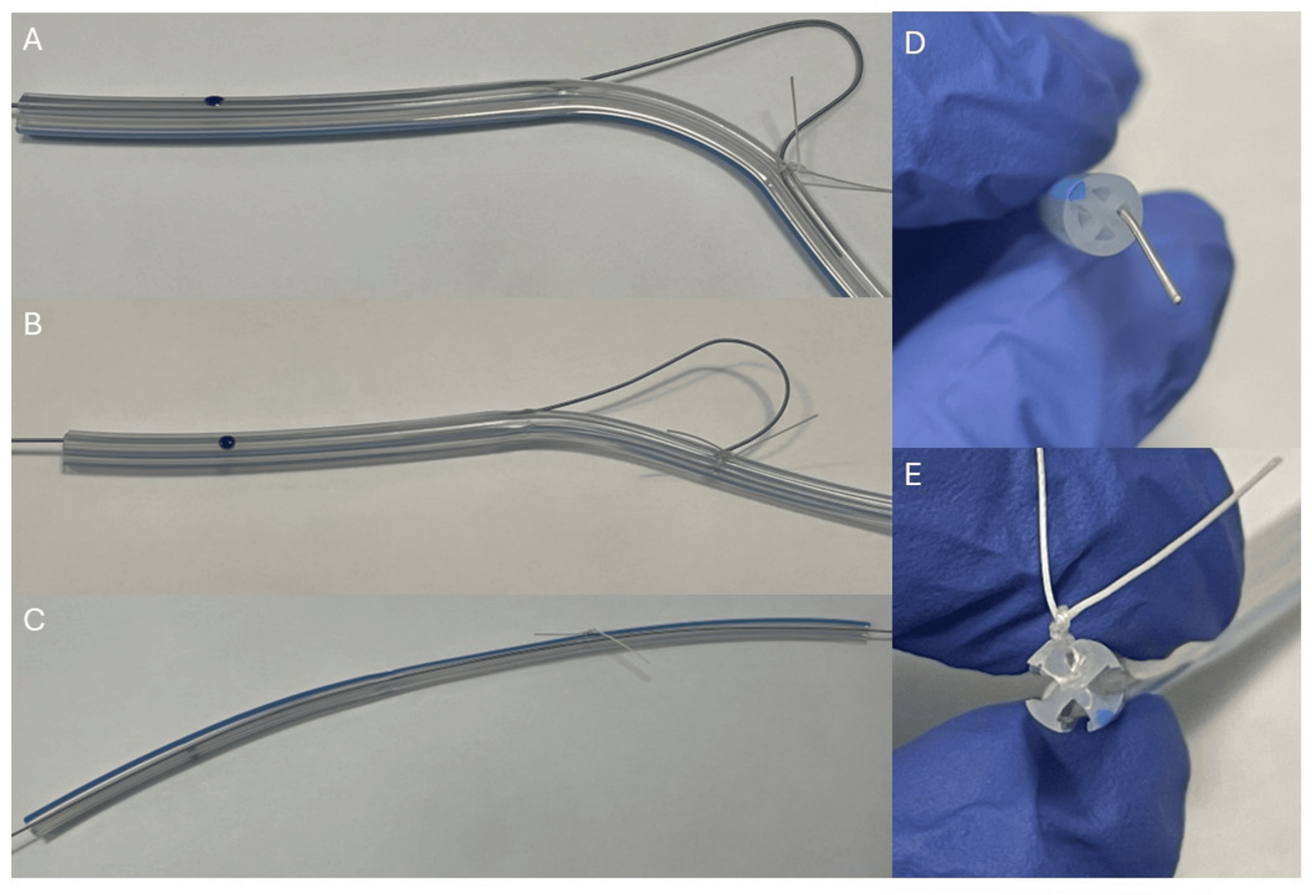
Manipulation of guidewire in tethered drain (A/B) Passage of the guidewire in the lumen is obstructed by the tethering suture. (C) Unobstructed passage of the guidewire as the lumen is not obstructed. (D) Close-up of the guidewire within the lumen at the distal end of the Blake drain. (E) Close-up of a suture that has pierced a portion of the Blake drain, closing off one of the four lumens. In this example, a 15-Fr Blake drain, 0.021-inch-diameter spring wire, and 2-0 vicryl, equivalent materials as mentioned in the article, were utilized.

Several methods have been described and investigated to avoid returning to the OR for the removal of retained sutures. Beshai et al. describe using a 17-Fr endoscopic visual urethrotome to localize intra-pelvic tethered Penrose drains and cutting the retaining sutures under direct visualization [7]. On the other hand, weighted traction was found to be inferior to manual traction for drain removal and also lead to increased drain retention in a cadaveric study [6]. The use of percutaneous interventional tools is not novel for the extraction of foreign bodies. A retained Jackson-Pratt drain fragment was removed with a 7-Fr balloon angioplasty catheter that was inflated from within [9]. A central venous catheter fragment was removed in a similar manner with a Fogarty catheter [10]. For our specific case, the patient had a Blake drain, which is different from Penrose and Jackson-Pratt drains due to the absence of a single lumen. Rather, it is a round silicone tube with four open lumens (Figure 3). These internal dimensions would hypothetically limit the application of some of the aforementioned non-traction techniques. With the largest guidewires, often less than 3 Fr in diameter, the channels of the Blake drain were easily assessed. While the case precludes an in situ examination, these images model the tethering suture and its interactions with the guidewire (Figure 4).

Interestingly, the vascular and cardiac literature validates the use of guidewire electrification. With the LAMPOON procedure, the anterior mitral leaflet is split via guidewire electrification to prevent left ventricular outflow tract obstruction prior to valve replacement [11]. Similar applications are used for preventing coronary obstruction during transcatheter aortic valve replacement and crossing aortic coarctation [12,13]. Of note, these techniques often employ the use of two catheters, whereby a snare is used to catch the guidewire, thus creating a protected electrified segment. In a hypothetical situation where a larger lumen drain is punctured by a fascial suture, a single guidewire may not be sufficiently obstructed and simply bypass the retaining suture; the use of dual wires with a snare could have an application in this scenario. In neurosurgical literature, a similar technique has been described for the removal of retained ventricular catheters. Chehrazi and Duncan first describe a method of pulling the retained catheter through an insulated suction tube that has been attached to an electrocautery unit to lyse adhesions [14]. Others have successfully performed adhesion resection with the safe removal of catheter through the application of current through catheter stylets and endoscopic monopolar wires [15-18].

There are several key limitations in our study. First, our primary objective is just the proposal of a novel method of drain removal as proof of concept. Our specific case was performed in a hybrid OR out of precaution that surgery could be performed in the setting of guidewire cauterization failure. In the future, it would not be unreasonable to perform this procedure in the interventional radiology (IR) suite without anesthesia, which would decrease the additional degree of risk for the patient. Thus, our results may not necessarily be generalizable at the current stage. Next, there are several variables that are not clear. How the tubing was tethered (pierced or incarcerated) and the caliber of the retaining suture are both unknown. It is also possible that the repeated insertion of the guidewires may have contributed to weakening or breaking the retaining suture. It has been found that drain retention rates did not differ between suture placements, while suture caliber affects its failure rate [6]. The procedure's potential for injury to neural elements, namely, the spinal cord, is not known, though direct fluoroscopic guidance facilitated general avoidance of the central canal.

## Conclusions

We report an original case and proof of concept of tethered drain removal via guidewire cauterization under fluoroscopic guidance. It utilizes commonplace equipment/materials and avoids re-opening the original wound for exploration, allowing the patient to avoid a longer hospital stay as well as minimizing the risk of wound infection. While additional investigation is warranted, this is a promising and approachable technique. Future studies should examine different types of drains, sutures, wires, cautery, as well as risk profiles in a cadaveric model.
